# The Relationship between Dynamic Balance, Jumping Ability, and Agility with 100 m Sprinting Performance in Athletes with Intellectual Disabilities

**DOI:** 10.3390/sports12020058

**Published:** 2024-02-14

**Authors:** Ghada Jouira, Dan Iulian Alexe, Dragoș Ioan Tohănean, Cristina Ioana Alexe, Răzvan Andrei Tomozei, Sonia Sahli

**Affiliations:** 1 Research Laboratory Education, Motricité, Sport et Santé (EM2S) LR19JS01, High Institute of Sport and Physical Education of Sfax, University of Sfax, Sfax 3000, Tunisia; jouiraghada0825@gmail.com (G.J.); sonia.sahli.isseps@gmail.com (S.S.); 2 Department of Physical and Occupational Therapy, “Vasile Alecsandri” University of Bacău, 600115 Bacău, Romania; 3 Department of Motric Performance, “Transilvania” University of Brașov, 600115 Brașov, Romania; 4 Department of Physical Education and Sports Performance, “Vasile Alecsandri” University of Bacău, 600115 Bacău, Romania; alexe.cristina@ub.ro; 5 Department of Physical Education and Sport, Alexandru Ioan Cuza, University of Iași, 700506 Iaşi, Romania; andrei.tomozei@uaic.ro

**Keywords:** intellectual disabilities, sprinting, dynamic balance, jumping ability, physical performance

## Abstract

Sprinting is a competitive event in athletics that requires a combination of speed, power, agility, and balance. This study investigated the relationship between dynamic balance, jumping ability, and agility with 100 m sprinting performance in athletes with intellectual disabilities, addressing an underexplored connection. A sample of 27 sprinters with intellectual disabilities participated in this study and completed 100 m sprint and various tests, including the Y Balance Test (YBT), the Crossover hop test, squat jump (SJ), countermovement jump (CMJ), and *t*-test to evaluate their dynamic balance, jumping ability, and agility, respectively. The findings revealed significant negative correlations between the YBT, Crossover hop test, SJ, and CMJ and 100 m sprint performance (r range: −0.41 to −0.79, *p* < 0.05). Regression analysis identified these variables as significant predictors (R^2^ = 0.69; *p* < 0.01). SJ exhibited the strongest association with 100 m sprint performance, (R^2^ = 0.62, *p* < 0.01). The agility *t*-test did not show a significant association. The combination of the YBT ANT and SJ demonstrated a predictive capability for 100 m sprint performance (R^2^ = 0.67, *p* < 0.001). In conclusion, this study revealed predictive capabilities between dynamic balance, jumping ability, and 100 m sprint performance in sprinters with intellectual disabilities.

## 1. Introduction

The prevalence of intellectual disability, characterized by cognitive limitations and deficits in communication, socialization, and self-care skills, affects approximately 200 million people, representing 2.6% of the global population with an intelligence quotient (IQ) below 70 [1,2]. The Special Olympics, one of the most popular recreational programs for individuals with intellectual disabilities, offers a platform for breaking barriers and pushing limits through sports, attracting over 4.4 million participants from over 180 countries in 32 Olympic-style individual and team sports [3]. Among these sports, track and field stands out as a common discipline among individuals with intellectual disabilities [4,5]. Sprinting is an important component of several track and field events. Sprinters with intellectual disabilities face unique challenges in their athletic pursuits. In addition to the cognitive impairments [1], these individuals often have physical limitations [6,7] that affect their ability to perform at their best. Sprinting is a complex motor skill that involves the coordination of various physiological and biomechanical factors, including jumping ability, change in direction, and dynamic balance. For athletes with intellectual disabilities, impairments in these factors can impact their sprint performance and obstruct their ability to execute proper sprinting technique.

Sprinting involves rapid acceleration and maximal speed running, which require explosive power and strength. Jumping ability is an important factor in sprint performance, as it involves the explosive power that is required for the initial acceleration phase of sprinting [8]. Forces that are applied during ground contact, especially in jumping, have been shown to correlate with the ability to attain maximum speeds [9]. This force that is generated in jumping can be translated into sprinting, enabling athletes to exert greater force during push-off and cover more distance per stride [9,10]. This can result in faster sprinting times and better overall performance. In fact, jumping involves the use of several muscle groups, including the quadriceps, glutes, calves, and core muscles, which are also used during sprinting [10]. Enhancing one’s jumping ability can result in heightened lower body strength and explosive power, which are essential for sprinting events. Studies have shown a strong correlation between jumping performances and the speed that is achieved in 10 m, 30 m, 50 m, and 100 m sprints among high-level typically developed sprinters [11,12], suggesting that increasing jumping is fundamental when attempting to improve sprint performance. Moreover, agility is a crucial component in most field sports [13]. Agility refers to the ability to move quickly and change direction with ease, balance, and control [13]. In sprinting, agility allows athletes to adjust their body position and change direction rapidly, enabling them to avoid obstacles, evade defenders, or make quick turns around a track [14]. The ability to change direction quickly can also improve an athlete’s acceleration and deceleration, which can be critical in sprinting events and reduce injury risks in typical development athletes [15].

Dynamic balance refers to the ability to maintain balance while in motion, such as when changing direction or making sudden movements [16]. Dynamic balance is important for sprinting tasks, as athletes must maintain balance and control at high speeds while accelerating, decelerating, and changing direction. Good dynamic balance allows sprinters to maintain proper running form and technique, which is essential for maximizing their speed and power [17]. However, it has been found that typical development athletes with poor dynamic balance experience a loss of control, which can lead to inefficient movements and slower times. Improving one’s dynamic balance can also reduce the risk of injury in runners with and without intellectual disabilities [18]. Previous research suggested that athletes with intellectual disabilities exhibit impaired postural balance when compared to the general population [19]. This deficit has been attributed to the deficiencies in visual [20], proprioceptive [21], and vestibular [22] inputs. The compromised postural balance in athletes with intellectual disabilities may heighten their vulnerability to lower extremity injuries, acknowledged as a key risk factor across various sports in typically developing athletes [23]. Consequently, the observed reduction in postural balance not only impedes athletes’ sprint performance but also amplifies the probability of injury occurrence [18].

As far as we know, no previous research has examined the relationship between jumping, agility, dynamic balance, and sprinting performance among athletes with intellectual disabilities. Understanding the relationship between these variables could provide important insights into the unique physical challenges that are faced by sprinters with intellectual disabilities and help develop more effective training programs, tailored to this population. The objective of this research is to explore the relationship between dynamic balance, jumping ability, and agility with sprinting performance in sprinters with intellectual disabilities. The hypothesis is that dynamic balance, jumping, and agility performances are significantly associated with sprint performance in athletes with intellectual disabilities.

## 2. Materials and Methods

### 2.1. Participants

The present study involved a group of 27 male athletes from a special educational center who exhibited mild intellectual disabilities, determined by their IQ scores falling within the range of 50 to 70 on the Wechsler Adult Intelligence Scale–Fourth Edition test [24], with the average IQ for individuals without intellectual disabilities typically exceeding 70. Details regarding their demographics and anthropometrics were extracted from medical records, evaluated by the center’s psychologist, and cross-verified with inputs from their coach (see Table 1). Participants in our study met the inclusion criteria of being male with a mild intellectual disability, a middle socio-economic status, and sprinting experience between 4 and 6 years. All of the athletes regularly attended training sessions at the National Athletics Stadium. They participated in these sessions four times a week, with each session lasting two hours. The exclusion criteria were carefully defined to exclude individuals with co-morbid conditions, visual and/or vestibular disorders or diseases, lower limb or lower back injuries, neuromuscular disorders, muscle problems, physical conditions, multiple disabilities, and current medication use. Confirmation of these criteria was obtained through a thorough review of their medical history files. These exclusion criteria aimed to minimize confounding variables that could influence the study outcomes. Prior to their participation, the athletes, along with their parents and coach, were provided with a detailed explanation of the potential risks and benefits associated with the study. Following this, athletes provided verbal consent, while written consent was obtained from their parents or coach. The present study was conducted according to the Declaration of Helsinki, and the protocol was fully approved by the local Committee of Protection of Persons (C.P.P.SUD N 0228/2020).

### 2.2. Study Design

**Figure 1:**
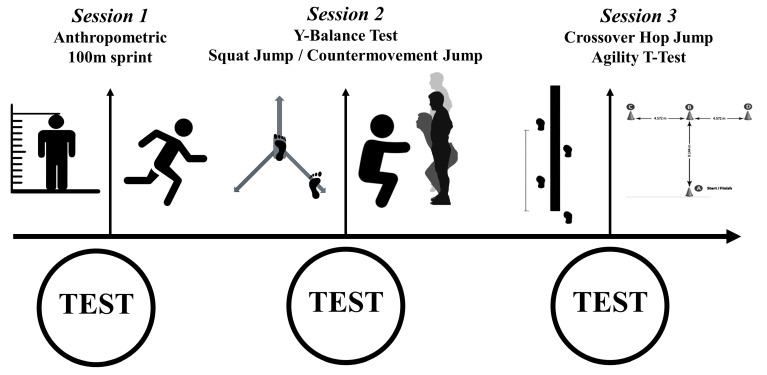
Schematic representation of the testing sessions.

The aim of the present study was to explore the relationship between jumping ability and agility in 100 m sprint performance in athletes with intellectual disabilities. To achieve this, a cross-sectional correlational design was used. Dynamic balance was evaluated through the Y Balance Test (YBT) and Crossover hop test. Jumping tests were assessed using squat jump (SJ) and countermovement jump (CMJ). Agility was evaluated using *t*-test. Participants completed three testing sessions (Figure 1). In the first testing session, anthropometric data were measured and included. The order of tests in our study was organized to account for factors such as potential fatigue, the specific demands of each assessment, and the nature of the tests. The second session focused on dynamic balance (YBT) and jumping ability (SJ and CMJ), intentionally separated from the third session to prevent fatigue during single-leg assessments. Placing the Crossover hop test and agility *t*-test in the third session aimed to complement earlier tests and minimize cumulative fatigue, recognizing the field testing nature of the agility *t*-test. To accommodate circadian variability, all testing sessions were conducted at the same time of day and aligned with participants’ regular training times. The assessments took place under consistent weather conditions (29 °C and 50% humidity). All participants were cooperative and enthusiastic, and they were well versed in the testing procedures to minimize any effects that may have occurred due to a learning curve.

### 2.3. Measurements

#### 2.3.1. The 100 m Sprint Performance

The 100 m sprint test requires covering a distance of 100 m, emphasizing maximum acceleration before crossing the starting line, with time recorded. The test was conducted in the athletic stadium where the participants performed their habitual training sessions. All athletes were given an adequate warm-up and practice first, as well as some encouragement to continue running hard past the finish line. The initiation position was standardized, commencing from a fixed stance with one foot positioned behind the starting line, devoid of any rocking movements. In order to measure the time of each participant’s performance, a stopwatch was used. Sprinters performed 2 attempts of 100 m test to assess the maximum speed, with a 5–7 min interval between attempts [25].

#### 2.3.2. Dynamic Balance Tests

Dynamic balance was evaluated through the YBT and Crossover hop test.

The YBT requires the athlete to achieve balance on a single leg, with the foot positioned at the center of the grid. Simultaneously, the athlete must extend the other leg as far as possible in three specific directions: anterior (ANT), posterolateral (PLAT), and posteromedial (PMED). The examiner recorded the distances reached by the athlete by marking the tape measure at the point where the athlete’s reach came to an end. The YBT composite score is calculated by adding the three reach distances and normalizing the outcomes to the length of the lower limb. The distance for each reach is divided by the leg’s length (from anterior superior iliac spine to medial malleolus) and then multiplied by 100. The test involves three attempts for each condition on both the right and left legs, with a 2 min resting period allowed between attempts. The greatest successful reach for each direction was used for analysis. This test is valid and reliable in athletes with intellectual disabilities [26].

The participants executed the Crossover hop test in a series of three consecutive attempts, spanning a 15 cm line marked on the floor to ensure standardized and precise execution. In this test, the participant was tasked with jumping on one leg and subsequently landing on the same leg that was used for propulsion. Test instructions specified that participants should place their hands on their hips, sustain the landing position for 3 s, and refrain from losing balance or making additional movements with the free limb. The distance achieved by the participant was measured in meters from the take-off line to the heel in the final position. Two attempts were made, and the best distance was recorded. This test is valid for athletes with intellectual disabilities [27].

#### 2.3.3. Jumping Tests

The SJ and CMJ were performed using the Optojump plate (Microgate, Bolzano, Italy), which determined jump height (h) based on flight time (t) and acceleration due to gravity (g) using the formula h = t^2^ × g/8 [28]. In the SJ, the sprinter initiated from a static position, hands on hips, maintaining a 90° knee flexion angle for 2 s before each trial, with no preparatory movements. Regarding the CMJ, the sprinter was instructed to position their hands on their hips to eliminate any influence of arm movements on vertical jump performance. He executed a downward movement, followed by full extension of the lower limbs. In both tests, he aimed to achieve maximum jump height. Precautions were taken to ensure proper technical execution, such as maintaining extended legs during flight time. The best trial in terms of SJ and CMJ height was used for further analysis. Three attempts were made, with a rest period of about 2 min between attempts. These tests are valid in athletes with intellectual disabilities [29].

#### 2.3.4. Agility Test

To assess agility, the *t*-test was administered, which involved measuring the speed of covering distances while changing directions in a side shuffle, sprinting forward, and running backwards. Prior to the test, athletes received clear instructions emphasizing the importance of performing the backward running component with caution. To perform the test, four cones were arranged in a T shape, with cone B placed 9.14 m from the starting cone A, and two additional cones, C and D, positioned 4.57 m on either side of cone B. Participants were instructed to sprint forward 9.14 m from cone A to cone B, touch it with their right hand, shuffle 4.57 m to the left to cone C, touch it with their left hand, then shuffle 9.14 m to the right to cone D and touch it with their right hand. Subsequently, they were to shuffle 4.57 m back to the left to cone B, touch it with their left hand, before finally running back to cone A. Participants were allowed to take a quick look during the running back phase to maintain situational awareness without compromising safety. A chronometer was used to measure the time taken to complete the test. Each participant performed the test twice, and their best time was recorded in seconds [30]. This test is valid in athletes with intellectual disabilities [31].

### 2.4. Statistical Analysis

Statistical analysis was conducted using SPSS 25 (SPSS Inc., Chicago, IL, USA). Means and standard deviation (SD) were computed for each variable (Table 2). The 95% confidence interval (CI) was calculated for the means. Data normality was assessed with the Shapiro–Wilk test. A paired *t*-test was conducted to assess the differences between the right leg and left leg in the YBT across all directions and the composite score. Pearson’s correlation coefficient (r) was utilized to identify correlations, with the following thresholds: <0.1, trivial; <0.1–0.3, small; <0.3–0.5, moderate; <0.5–0.7, large; <0.7–0.9, very large; and <0.9–1.0, almost perfect [32]. The association between YBT results (ANT, PLAT, PMED directions, and the composite score), SJ, CMJ, and the Crossover hop test and agility *t*-test as independent variables, and the 100 m sprint test result as the dependent variable, was assessed through linear regressions. Regression *p*-values and R^2^-values were calculated. R^2^ represents the percentage of total variation in the dependent variable, explained by the independent variable. An R^2^ of 1.0 indicates a perfect fit, while any value below 1.0 suggests that some variability in the data is not accounted for by the model [33]. The significant *p*-value was set at 0.05.

## 3. Results

The means (SD) and 95% CI for the measured variables are presented in Table 2. The paired *t*-test indicated no significant differences between the right and left legs in any YBT direction or composite score (*p* > 0.05). Consequently, we proceeded with the right leg for the remaining analyses.

The Shapiro–Wilk test was employed to check the normality of the data. Table 3 presents the Pearson correlations between the YBT, Crossover hop test, SJ, CMJ, and agility *t*-test with 100 m sprint performance. The findings revealed that the YBT composite score had the strongest negative correlation with 100 m sprint performance considering data from YBT, followed by YBT PMED, YBT PLAT, and YBT ANT (Table 3). The Crossover hop test, SJ, and CMJ were also found to be significantly negatively correlated with 100 m sprint performance, with the strongest correlation among all independent variables being SJ (Table 3).

**Figure 2:**
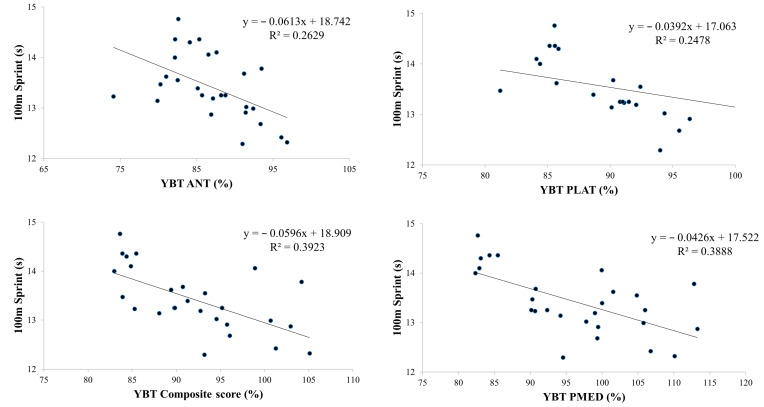
Partial regressions between the Y Balance Test (YBT) in anterior (ANT), posterolateral (PLAT), and posteromedial (PMED) directions and the 100 m sprint test.

**Figure 3:**
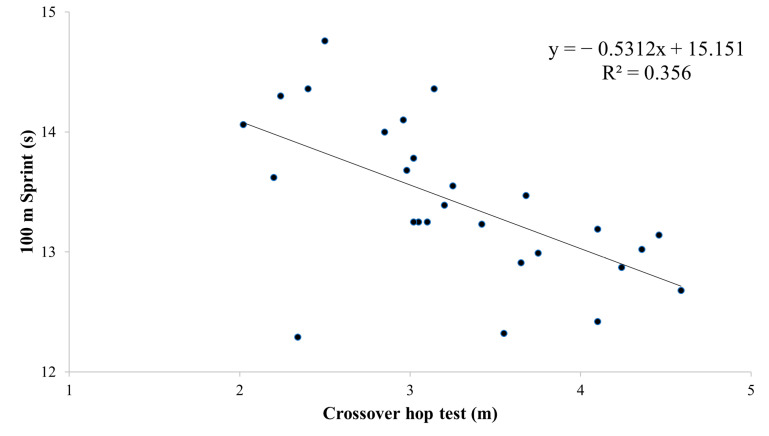
Partial regressions between the Crossover hop test and the 100 m sprint test.

**Figure 4:**
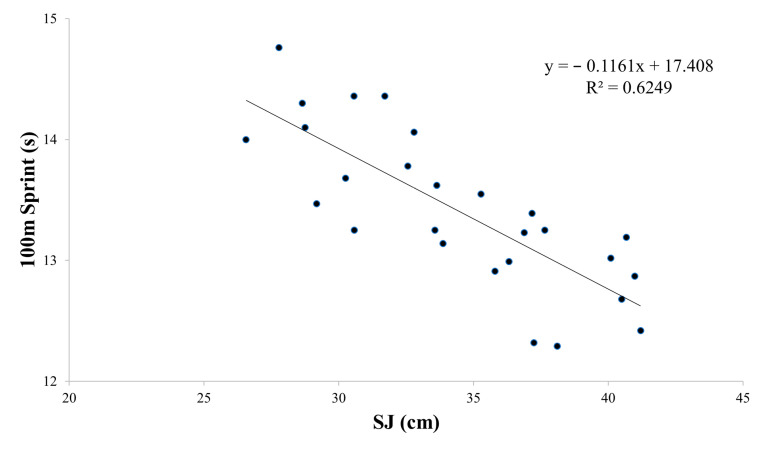
Partial regressions between the squat jump (SJ) test and the 100 m sprint test.

**Figure 5:**
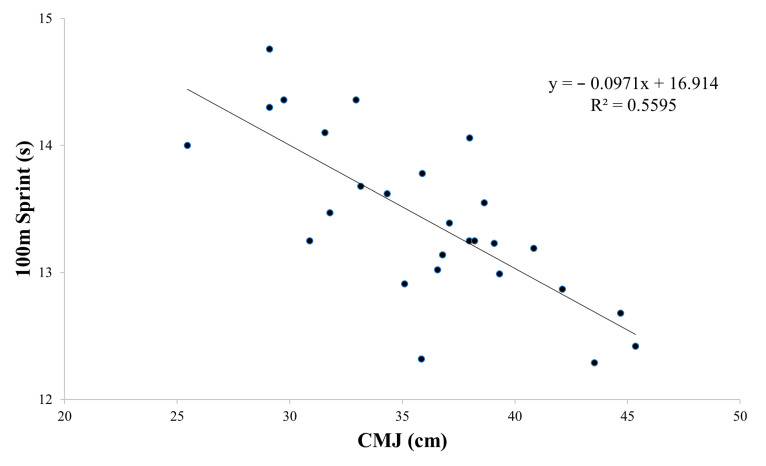
Partial regressions between the countermovement jump (CMJ) test and the 100 m sprint test.

**Figure 6:**
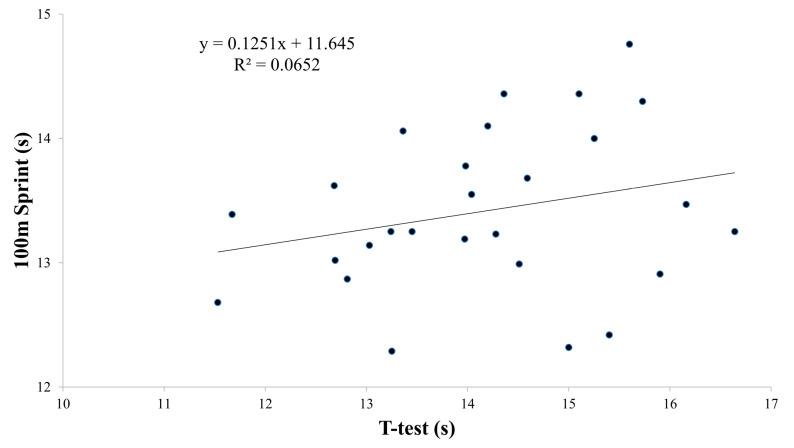
Partial regressions between *t*-test and the 100 m sprint test.

Based on the findings presented in Table 4 and Figure 2, Figure 3, Figure 4 and Figure 5, the physical tests (YBT, Crossover hop test, SJ, and CMJ) were significantly associated with 100 m sprint performance (B range: −0.044 to −0.097, *p* < 0.01). The analysis revealed a particularly strong association between SJ and 100 m sprint performance, with an R^2^ value of 0.62 (Figure 4). On the other hand, the agility *t*-test was not found to have a significant association with 100 m sprint performance (Table 4, Figure 6).

The regression model had a good fit to the data, as indicated by a high R^2^ value of 0.69, which indicates that the model explains 69% of the variance in 100 m sprint performance (Table 4). The adjusted R^2^ value of 0.57 suggests that approximately 57% of the variability in the outcome variable can be explained by the independent variables that are included in the model, after accounting for the number of predictors and sample size.

The results of Table 5 demonstrate that the combination with the highest adjusted R^2^ is the model that includes the variables YBT ANT and SJ. This model demonstrates a strong predictive capability for 100 m sprint performance, with R^2^ = 0.67.

## 4. Discussion

The aim of this study was to explore the relationship between dynamic balance, jumping ability, and agility in sprinting performance in sprinters with intellectual disabilities. The Pearson correlation analysis showed a significant negative correlation between the 100 m sprint test and several physical tests, including the Crossover hop test (right and left legs) and YBT (right and left legs) as dynamic balance tests and SJ and CMJ tests as jumping test. However, no significant correlation was found between the 100 m sprint test and the agility *t*-test. Furthermore, the regression model showed that dynamic balance and jumping tests significantly predicted the 100 m sprint performance in sprinters with intellectual disabilities. These findings suggested that an individual’s ability to balance and stabilize on one leg, as well as their explosive power, are important factors that influence sprinting performance in sprinters with intellectual disabilities.

To the best of our knowledge, no study has been conducted to investigate the potential correlation between dynamic balance and 100 m sprint performance in sprinters with intellectual disabilities. One potential explanation for the relationship between dynamic balance and 100 m sprint performance is that dynamic balance might play a role in elevating an athlete’s capacity to generate force, specifically during the critical push-off stage of a sprint, thereby enhancing stability and control in the lower extremities. This may allow the athlete to maintain an optimal body position and generate more force during each stride, which can lead to improved sprinting performance [34]. The YBT goes beyond assessing an athlete’s ability to maintain balance; it also evaluates an individual’s muscle coordination performance, considering the intricate biomechanics of hip and ankle joint movements [35,36]. In the context of a 100 m sprint, the efficiency of joint movements, particularly at the hip and ankle levels, is crucial throughout its phases [37,38]. Therefore, the observed negative correlation between dynamic balance, as assessed by the YBT, and sprint performance implies that athletes with a good dynamic balance may possess a biomechanical advantage. This advantage could lead to more efficient joint movements during various phases of a sprint, potentially contributing to improved sprinting performance. Moreover, YBT requires neuromuscular features, such as lower limb coordination, and strength [39]. These elements play a pivotal role in influencing an athlete’s performance during a 100 m sprint. In fact, the emphasis on lower limb coordination in the YBT suggests that athletes excelling in this aspect may exhibit more synchronized and efficient movements throughout the various phases of the sprint [17]. Furthermore, the integration of strength as a neuromuscular feature suggests that greater lower limb strength may contribute to enhanced force production during push-off, facilitating rapid acceleration in the 100 m sprint [17,40]. In addition, the Crossover hop test, incorporating both eccentric (landing) and concentric (push-off) muscle actions, establishes crucial links to sprinting performance. Sprinting relies significantly on both eccentric (muscle lengthening) and concentric (muscle shortening) phases, each serving essential roles [41]. The eccentric phase contributes to deceleration, joint stability, and force absorption, while the concentric phase is important for propulsion and acceleration [41]. Therefore, the Crossover hop test, by encompassing both phases that are crucial to sprinting, may provide a comprehensive evaluation of the varied demands of sprinting.

It is important to note that dynamic balance may also be important for sprinting performance because it can help prevent injuries. In fact, sprinting involves rapid and oscillatory movements of the center of gravity, as well as an instability caused by the body’s translation, limb angular momentum, and vertical or oblique displacement of the pelvis [42]. As a result, dynamic balance is crucial to prevent falling while running at high speeds, as the center of gravity shifts over the support foot during the process [17]. Moreover, maintaining good dynamic balance during high-speed movements can reduce the risk of ankle and knee injuries, which can be common in sprinters with intellectual disabilities [18].

In addition, the present study demonstrated that jumping tests are a reliable predictor of 100 m sprint performance in sprinters with intellectual disabilities. This finding is consistent with previous research in athletes without intellectual disabilities [8,12,43,44], suggesting that the relationship between jumping ability and sprinting performance is not limited to individuals without disabilities. In fact, jumping and sprinting require similar muscle groups (quadriceps, hamstrings, and gluteal muscles) and neuromuscular activation patterns, and improving one can lead to improvements in the other [11,12]. Specifically, jumping ability is closely linked to lower extremity power and explosiveness, which are essential for accelerating and maintaining high speeds during sprinting [44]. In the context of sprinting, the CMJ and SJ tests are valuable tools for assessing various aspects of explosive strength, each capturing distinct characteristics of the stretch–shorten cycle. The CMJ’s emphasis on the cycle of stretching and shortening aligns with the dynamic nature of sprinting, where rapid, repetitive cycles of muscle stretching and contraction occur with each stride. This allows the athlete to use elastic energy efficiently, contributing to the explosive thrust that is crucial for sprint acceleration and maintaining high speeds [45]. In contrast, the SJ, which emphasizes immediate force production without the stretch–shortening cycle, replicates the explosive push-off from a static starting position, resembling the initial burst during a sprint [46]. Both tests, therefore, offer unique insights into the facets of explosive strength that are relevant for different phases of sprinting. In typical development athletes, vertical and horizontal jump tests, such as SJ, CMJ, and Crossover hop, have been employed as predictors of sprint performance, with higher jumping scores correlating with faster sprint times [11,12]. This relationship is attributed to the shared requirement for a high force production in a short amount of time in both activities [12]. Jumping tests measure maximal force production in a single effort, while sprinting demands the ability to produce high levels of force repeatedly over a short period. Consequently, athletes with superior jumping ability may also possess a greater capacity for producing high levels of force quickly, leading to faster sprint times. Moreover, jumping tests mirror the movement pattern of sprinting, emphasizing lower extremity power and explosiveness [11].

However, the study results showed no significant correlation between the agility *t*-test and 100 m sprint performance in sprinters with intellectual disabilities. The lack of a significant correlation between the agility *t*-test and sprint performance may be due to the fact that agility is a separate skill that requires different neuromuscular and cognitive abilities to sprinting [13]. Agility involves quick changes in direction, acceleration, and deceleration [13], while sprinting involves running at maximal speed over a straight line. In addition, in the *t*-test, running backwards involves a distinct motor skill compared to forward running. When an athlete runs forward, the motor patterns and coordination involve pushing off the ground with the toes, propelling the body forward, and maintaining balance during forward propulsion. Running backwards requires a reversal of these motor patterns. The athlete must push off backwards, coordinating movements that involve lifting the heels rather than the toes and moving in the opposite direction [47]. This demands a different set of neuromuscular coordination, spatial awareness, and proprioceptive skills [47]. Importantly, athletes with intellectual disabilities may have limitations in their ability to process information and react quickly to visual and auditory stimuli [48], which can also impact their agility performance. On the other hand, the sprinting ability in athletes with intellectual disabilities may be influenced by factors such as muscle strength, power, and endurance, which may be less affected by their cognitive impairments [49,50]. Therefore, while agility may be an important component of overall athletic performance, it may not be a reliable predictor of sprinting ability in athletes with intellectual disabilities.

This study has some limitations. In fact, the exclusive focus on male athletes with mild intellectual disabilities, potentially restricts the generalizability of the findings to broader populations. As a future line of research, it may enhance the generalizability of the study to consider a more diverse sample, including both sexes and individuals with various types of intellectual disabilities. Furthermore, the present study only assessed sprint performance over a distance of 100 m, which may not be representative of all types of sprinting events or sports. Future studies could explore a broader range of sprint distances and sports disciplines to provide a more comprehensive understanding of the relationship between dynamic balance, jumping ability, and agility with sprint performance across diverse athletic contexts. In addition, the study only examined the relationships between the tests used in the study and sprint performance and did not investigate the causal mechanisms underlying these relationships. Therefore, it is not clear whether improvements in lower limb power and stability directly lead to improvements in sprint performance, or whether other factors may also be involved. Future studies may explore the mechanisms underlying the observed connections in this study for a more in-depth understanding.

Based on this study’s findings, coaches and trainers for athletes with intellectual disabilities are recommended to incorporate a range of exercises that target lower limb power and dynamic balance and coordination into training programs for athletes with intellectual disabilities. These may include plyometric exercises, balance training, and strength training for the lower body. Additionally, our findings underscore the SJ as a robust predictor of 100 m sprint performance in athletes with intellectual disabilities, suggesting a targeted avenue for inclusion in training programs that are focused on enhancing lower extremity power and explosiveness. Coaches and trainers are advised to prioritize exercises, including resistance training, plyometrics, and drills, that are designed to optimize force generation during the concentric phase of the jump. Furthermore, the combination of SJ and the YBT ANT appears to be particularly effective in predicting 100 m sprint performance in athletes with intellectual disabilities, revealing a synergistic effect. This highlights the need for a dual-focus training approach that addresses both explosive power (measured by SJ) and dynamic balance (measured by YBT ANT). Therefore, training programs should incorporate an overall strategy to improve athletes’ ability to generate explosive force while simultaneously improving stability and control. Such an integrated training approach aligns with the goal of optimizing athletes’ sprint performance.

## 5. Conclusions

In conclusion, our study highlighted the importance of dynamic balance and jumping tests in predicting 100 m sprint performance in athletes with intellectual disabilities. SJ was identified as a strong indicator, highlighting the need to address various aspects of lower extremity power in their training programs. The study notably demonstrated that the combination of the YBT ANT with SJ presented a remarkable predictive capacity. These results provide valuable information to trainers and coaches of sprinters with intellectual disabilities, highlighting the importance of targeted training interventions to improve both dynamic balance and lower extremity explosive power, thereby optimizing sprint performances in this population.

## Figures and Tables

**Table 1:** Means (SD) of the general characteristics.

General Characteristics	Means (SD)
Gender	Male
Age (years)	25.11 (3.71)
Height (cm)	168.43 (7.28)
Mass (kg)	65.87 (3.77)
BMI (kg/m^2^)	23.33 (1.9)
IQ	59.93 (5.73)
Type of training	100 m sprints
Training experience in years	5.84 (0.68)
Training Frequency (sessions/week)	4
Training volume (hours/session)	2
Right dominant leg	100%

Abbreviations: SD: standard deviation; BMI: body mass index; IQ: intelligence quotient.

**Table 2:** Means (SD) of the independent variables: Y Balance Test (YBT), squat jump (SJ), countermovement jump (CMJ), Crossover hop test, and agility *t*-test; and dependent variable: 100 m sprint performance.

Independent Variables	Means (SD)	95% Confidence Interval (CI)	
		Lower	Upper
Dynamic balance tests			
YBT ANT Right leg (%)	86.89 (6.52)	84.31	89.47
YBT PLAT Right leg (%)	93.39 (8.69)	90.95	97.83
YBT PMED Right leg (%)	97.69 (9.69)	93.85	101.52
YBT composite score Right leg (%)	92.99 (7.73)	90.01	95.97
YBT ANT Left leg (%)	86.94 (5.44)	84.79	89.09
YBT PLAT Left leg (%)	93.11 (8.69)	89.84	96.38
YBT PMED Left leg (%)	96.30 (9.51)	92.54	100.07
YBT composite score Left leg (%)	92.12 (6.83)	89.42	94.82
Crossover hop test (m)	3.27 (0.73)	2.97	3.55
Jumping tests			
SJ (cm)	34.38 (4.43)	32.63	36.13
CMJ (cm)	36.04 (5.01)	34.05	38.02
Agility test			
*t*-test (s)	14.16 (1.33)	13.63	14.68
Dependent variable			
100 m sprint (s)	13.42 (0.65)	13.15	13.67

Abbreviations: YBT: Y Balance Test; ANT: anterior; PLAT: posterolateral; PMED: posteromedial; SJ: squat jump; CMJ: countermovement jump.

**Table 3:** Pearson correlations between Y balance test (YBT), squat jump (SJ), countermovement jump (CMJ), Crossover hop test, and agility *t*-test and 100 m sprint performance.

	YBT ANT	YBT PLAT	YBT PMED	YBT Composite Score	Crossover Hop Test	SJ	CMJ	Agility *t*-Test
100 m sprint	−0.41	−0.55	−0.65	−0.61	−0.59	−0.79	−0.74	0.25
*p*-value	0.03	0.003	<0.001	<0.001	0.07	<0.001	0.001	0.71

Abbreviations: YBT: Y Balance Test; ANT: anterior; PLAT: posterolateral; PMED: posteromedial; SJ: squat jump; CMJ: countermovement jump.

**Table 4:** Summary of the multiple linear models carried out for physical tests: Y Balance Test (YBT), squat jump (SJ), countermovement jump (CMJ), Crossover hop test, and agility *t*-test as independent variables and the 100 m sprint performance as dependent variable.

Independent Variables	Dependent Variable	B	Standard Error	t	*p*-Value
YBT ANT (%)	100 m sprint (s)	−0.042	0.018	−2.29	=0.031
YBT PLAT (%)		−0.041	0.012	−3.29	=0.003
YBT PMED (%)		−0.044	0.010	−4.32	<0.001
YBT composite score (%)		−0.053	0.014	−3.87	=0.001
Crossover hop test (m)		−0.53	0.140	−3.71	=0.01
SJ (cm)		−0.116	0.018	−6.45	<0.001
CMJ (cm)		−0.097	0.017	−5.63	<0.001
Agility *t*-test (s)		0.120	0.095	1.32	0.19
R	0.83				
R^2^	0.69				
Adjusted R^2^	0.57				

Abbreviations: YBT: Y Balance Test; ANT: anterior; PLAT: posterolateral; PMED: posteromedial; SJ: squat jump; CMJ: countermovement jump.

**Table 5:** Combination regression models for predicting 100 m sprint performance using Y Balance Test (YBT), squat jump (SJ), countermovement jump (CMJ), Crossover hop test, and agility *t*-test as independent variables.

Model	Dependent Variable	Independent Variables	R^2^	Adjusted R^2^	R	*p*-Value
1	100 m Sprint Performance	YBT composite score and Crossover	0.55	0.51	0.74	<0.001
2		YBT composite score and SJ	0.65	0.62	0.80	
3		YBT composite score and CMJ	0.59	0.56	0.77	
4		YBT ANT and Crossover	0.49	0.45	0.70	
5		YBT ANT and SJ	0.67	0.64	0.82	
6		YBT ANT and CMJ	0.62	0.58	0.78	
7		YBT PLAT and Crossover	0.48	0.44	0.69	
8		YBT PLAT and SJ	0.63	0.60	0.79	
9		YBT PLAT and CMJ	0.56	0.53	0.75	
10		YBT PMED and Crossover	0.53	0.49	0.73	
11		YBT PMED and SJ	0.64	0.61	0.80	
12		YBT PMED and CMJ	0.58	0.55	0.76	
13		Crossover hop and SJ	0.64	0.61	0.80	
14		Crossover hop and CMJ	0.62	0.59	0.78	
15		CMJ and SJ	0.63	0.60	0.70	

Abbreviations: YBT: Y Balance Test; ANT: anterior; PLAT: posterolateral; PMED: posteromedial; SJ: squat jump; CMJ: countermovement jump.

## Data Availability

The datasets used and/or analyzed during the current study are available from the corresponding author upon reasonable request.
